# The degradation of glycosaminoglycans by haloarchaea is apparently a common feature in hypersaline habitats worldwide

**DOI:** 10.3389/fmicb.2026.1846936

**Published:** 2026-05-29

**Authors:** Dimitry Y. Sorokin, Violetta La Cono, Laura Marturano, Gina La Spada, Francesca Crisafi, Francesco Smedile, Manuel Ferrer, Alexander G. Elcheninov, Ksenya S. Zayulina, Pham Duc Thinh, Ninh Khắc Bản, Carla C. C. R. de Carvalho, Elena A. Selivanova, Enzo Messina, Michail M. Yakimov

**Affiliations:** 1 Winogradsky Institute of Microbiology, Research Centre of Biotechnology RAS, Moscow, Russia; 2 Institute of Polar Sciences, ISP-CNR, Messina, Italy; 3 Instituto de Catalisis y Petroleoquimica (ICP), CSIC, Madrid, Spain; 4 Nha Trang Institute of Technology Research and Application, NITRA-VAST, Nha Trang, Vietnam; 5 Institute of Marine Biochemistry, IMBC-VAST, Hanoi, Vietnam; 6 Department of Bioengineering, Instituto Superior Técnico, Institute for Bioengineering and Biosciences, Universidade de Lisboa, Lisbon, Portugal; 7 Orenburg Federal Research Center, Institute for Cellular and Intracellular Symbiosis, Ural Branch of RAS, Orenburg, Russia; 8 Institute for Marine Biological Resources and Biotechnology, IRBIM-CNR, Messina, Italy

**Keywords:** extremely halophilic archaea, glycosaminoglycans, hyaluronic acid and chondroitin, hypersaline lakes and solar salterns, polysaccharide lyases and glycoside hydrolases

## Abstract

Recent extensive studies of the functional diversity of extremely halophilic archaea (haloarchaea) have significantly altered the long-held view of this unique group of extremophiles as halophilic heterotrophs utilizing either rich mixtures of amino acids or simple soluble monomeric compounds such as sugars or organic acids. Our recent efforts clearly demostrated that many haloarchaeal species possess significant hydrolytic potential for decomposition of various groups of difficult-to-degrade polysaccharides. However, so far, there was no evidence that these archaea can utilize acidic polysaccharides. In this study, we characterized three strains of haloarchaea isolated from brine and sediment samples from various regions of Eurasia, namely, hypersaline lakes of the Kulunda Steppe in Russia, marine solar salterns of Samouco in Portugal and Trapani in Italy, and salt production fields of Nha Trang and Cam Ranh in Vietnam, using hyaluronic acid (HA) as the sole carbon and energy source as an enrichment substrate. The isolates utilizing this glycosaminoglycan (GAG) were identified as new-species members of the genera *Natronoarchaeum* and *Haloarcula*. Apart from the HA they can also grow with another GAG - chondroitin sulfate A. Genome analysis of these strains identified the genes potentially encoding the enzymes responsible for their GAG-degrading activity and determined the origin of these genes. This type of metabolism was unknown in haloarchaea prior to this study, potentially allowing haloarchaea to thrive on a rather specific polysaccharides, uncharacteristic of hypersaline ecosystems. These results further confirm that haloarchaea represent a unique group of extreme halophiles with incredibly extensive metabolic capabilities, representing a vast, largely unknown reservoir of functional diversity and having strong ecological significance in carbon cycling in hypersaline ecosystems worldwide and promising biotechnological potential.

## Introduction

Extremely halophilic archaea (haloarchaea), currently classified within the class *Halobacteria*, phylum *Halobacteriota* (Chuvochina et al., 2023; Göker and Oren, 2024), thrive as the dominant group in prokaryotic communities inhabiting various types of salt-saturated terrestrial brines, such as natural hypersaline (a)thalassohaline lakes, marine solar salterns, crystallizer ponds, and salt production fields. Unlike other, archaea, haloarchaea are better known as primarily aerobic copiotrophs, capable of utilizing either rich mixtures of peptides and amino acids or simple soluble monomeric compounds such as sugars and organic acids (Oren, 2024). Our recent studies of the functional microbiology of hypersaline ecosystems across the Eurasian continent have focused on identifying haloarchaea with previously undetected metabolic potentials, one of which was the ability to utilize various recalcitrant polysaccharides (PSs) that may be present in these ecosystems. This has resulted in the isolation of numerous polysaccharidolytic halo(natrono)archaea (Sorokin et al., 2015, 2018, 2019a, 2019b, 2022, 2023, 2024a, 2024b, 2024c; Elcheninov et al., 2023; La Cono et al., 2020, 2023; Reva et al., 2023; Tulenkov et al., 2025), significantly undermining the long-held view of these organisms as mere heterotrophs. Thus, it becomes evident that some of them also exhibit highly specialized metabolic activities, breaking down a variety of complex and recalcitrant PSs into simpler compounds, thereby playing a crucial role of primary hydrolytics in hypersaline habitats.

Given the specificity of life forms adapted to and thriving in such extreme conditions, one might expect to find only a few major types of PSs in hypersaline habitats: starch, derived from halophilic green alga of the genus *Dunaliella,* which are known to accumulate this *α*-glucan in quantities representing up to 70% of total cellular carbon (Pick and Avidan, 2017), and chitin, derived from brine shrimps and brine flies of the genera *Artemia* and *Ephydra*, respectively. Although not entirely relevant, the presence of other polysaccharides in salt-saturated environments should also be considered. This applies primarily to cellulose and hemicellulose, derived from fallen trees and the numerous artificial wooden structures often present in solar salterns and salt production fields. The ability of many haloarchaea to degrade α-glucans and grow on them as a sole source of energy and carbon is well documented (Moshfegh et al., 2013; Oren, 2024), while the discovery of chitino-, cellulo-, and xylanolytic haloarchaea is relatively recent (see Tulenkov et al., 2025 and references therein).

All of the above-mentioned PSs are composed of neutral sugars, but hypersaline ecosystems can also contain significant amounts of a completely different type of polysaccharides. These are acidic PSs, namely mucopolysaccharides or glycosaminoglycans (GAGs), which are ubiquitously present as major components of extracellular matrices in all tissues and organs of vertebrates, especially birds and mammals (Oiki et al., 2017). Notably, GAGs are virtually not synthesized by either invertebrates or unicellular microorganisms (Yamada et al., 2011; Senni et al., 2011). Since salt-rich habitats lack native vertebrates, the only potential source of this type of polysaccharides is the carcasses of migratory birds, such as flamingos, waders, and gulls, that have fallen into lakes. This seems quite reasonable, since the high density of microbial life in hypersaline habitats provides a food source for huge flocks (>10^6^ individuals) of these birds. Chemically, GAGs are a group of large linear acidic polysaccharides composed of repeating core disaccharide units formed by glucuronic and/or iduronic acid linked to an amino sugar, either *N*-acetylglucosamine (GlcNAc) or *N*-acetylgalactosamine (GalNAc), via *α*(1 → 4), *β*(1 → 3), or β(1 → 4) glycosidic linkages. The sulfate groups as well as the uronic acids result in the glycosaminoglycan chains having a negative charge. Five GAGs have been identified to date: hyaluronic acid (HA), chondroitin (CND), dermatan, heparin/heparan and keratan.

Currently, virtually nothing is known about the prokaryotes involved in the degradation of glycosaminoglycans in hypersaline environments. Only recently, a planctomycetes, classified as *Natronomicrosphaera hydrolytica* gen. Nov., sp. nov., was isolated from soda lakes and characterized as a highly specialized bacterium in utilizing HA (Sorokin et al., 2025). This study provides the first evidence of such metabolism in haloarchaea, presenting the results of the identification, isolation, and characterization of isolates capable of depolymerizing and growing on HA and CND, which are key components of various extracellular matrix structures in vertebrates, including, for example, aggrecan. This important and predominant proteoglycan in mammals and birds forms large hydrated structures that provide cartilage with the necessary swelling force and weight-bearing capacity, which are crucial for skeletal development. Thus, as mentioned above, the presence of this and other, GAG-containing compounds is expected in hypersaline habitats due to the presence of foraging bird flocks there, and the decomposition of glycosaminoglycans is of certain ecological interest, which remains completely unexplored to date. Therefore, we sought to investigate the presence of GAG-utilizing halophiles in hypersaline ecosystems worldwide. To obtain initial enrichment cultures, brines and sediments from five different regions of Eurasia, from Portugal to Vietnam, were used. Using sodium hyaluronate with different molecular weights (50,000 or 2,000,000 Da) as the only source of carbon and energy resulted in selective enrichment across all samples. Three enrichment cultures, originating from the Kulunda salt lakes (Altai, Russia), Trapani solar salterns (Italy), and Nha Trang salt production fields (Vietnam), were further used for the further microbiological study, which led to the isolation of pure cultures of halophilic archaea belonging to the genera *Natronoarchaeum* (H-hyl1, Kulunda) and *Haloarcula* (H-hyl2, Italy and H-hyl6, Vietnam), capable of utilizing these polymers as growth substrates. All isolated strains were capable of degrading and growing, albeit to a lesser extent, on chondroitin sulfate A. In addition, the polysaccharide lyase activity of the three strains towards HA and CND was measured. *De novo* sequencing of the genomes of these strains allowed us to identify the genes encoding the enzymes responsible for their polysaccharidolytic activity and determine the origin of these genes. The two remaining enrichment cultures, originated from solar salterns of Samouco (Portugal) and freshly harvested salt of Cam Ranh (Vietnam), were used for molecular studies of the diversity of the GAG/degrading enzyme machinery. These results once again demonstrate the significant functional diversity of haloarchaea specializing in decomposition of polysaccharides and possessing an enzymatic repertoire sufficient to degrade various difficult-to-degrade polymers under hypersaline conditions.

## Experimental **procedures**

### Samples, enrichments and growth conditions

The upper oxic layer of sediment (top 3 cm), brine and freshly harvested salt samples were obtained from five hypersaline habitats with circum-neutral pH: (i) Samouco solar salterns, Alcochete, Portugal (N 38.7356635 E − 8.9976888); (ii) Trapani marine solar salterns, Western Sicily, Italy (N 37.980528 E 12.495000); (iii) Kulunda Steppe hypersaline lakes, Altai, Russia (N 51.690000–750,000 E 79.720000–88,000); (iv) Hon Khoi salt fields, Nha Trang, Vietnam (N 12.539860 E 109.208740); (v) Cam Ranh, Vietnam (N 11.921439 E 109.159131) (Supplementary Figure S1). Based on knowledge of the successful isolation and maintenance of the chitino- and xylanolytic haloarchaea (La Cono et al., 2020, 2023), the liquid neutral base mineral medium with following composition was used (g l^−1^): 230 NaCl, 5 KCl, 0.2 NH_4_Cl, 2.5 K_2_HPO_4_. The pH was adjusted to 7.4 by the addition of 1 M KOH. After sterilization (121 °C, 20 min) and cooling, the medium was supplemented with 950 mg l^−1^ MgCl_2_, 1 mL l^−1^ acidic trace metal solution and 1 mL l^−1^ vitamin mix (Pfennig and Trüper, 1992). Finally, 0.5 g l^−1^ of 2,000,000 Da sodium hyaluronate (Biosynth, Bratislava, Slovakia) or medium size HA50 (Mw 50,000–350,000 Da), provided by Evonik Industries AG (Essen, Germany), from 2% (w/v) stock solution was added as the carbon and energy source. The 50 KDa stock was filter-sterilized and kept at 4 °C, while the higher MW HA solutions were prepared on autoclaved distilled water and kept frozen before use as they were two viscous for filtration and also too heat-sensitive for autoclaving. The bacteria-specific antibiotics vancomycin and streptomycin were added (100 mg l^−1^ of each, final concentration) to prevent the growth of any halophilic bacteria. For initial enrichments, when possible, the combined sediment/brine inoculum (5% vol/vol) was added to the liquid neutral base mineral medium supplemented with hyaluronan. The enrichments from Kulunda Steppe were incubated at 35 °C on a rotary shaker at 120 rpm in 115 mL serum bottles closed with rubber stoppers with the total liquid volume of 30 mL. Other enrichments were cultivated statically at 40 °C in the same type of bottles. The incubation time varied from 1 to 3 months. Appearance of red coloration was an indication of growth of haloarchaea which was further confirmed by microscopy. The isolation strategy for pure hyaluronanolytic cultures consisted of several rounds of decimal-dilution transfers (each inoculation to fresh neutral base mineral medium with total salinity 240 g l^−1^) being a 10-fold dilution by adding 10% of culture to 90% of fresh medium (vol/vol), followed by a three-fold repetition of serial dilution-to-extinction up to 10^−9^ in the same medium. After the appearance of an intense red color, 5 μL of the grown culture was diluted in 5 mL of liquid medium, and then 5 μL of the resulting dilution was spread on agar plates (1.5% w/vol) supplemented either with the 2 MDa or 50 KDa HA (0.1% vol/vol). The grown colonies were picked and inoculated the liquid medium that had been supplemented with both types of HA. Finally, the purity of isolates was checked by 16S rRNA gene cloning and sequencing of at least 10 clones from each library and, finally, by the whole genome sequencing (as many haloarchaeal genera have several divergent intraspecific rrn operons).

### Polysaccharide utilization activity and chemical analyses

The primary indicator of the use of HA and CND was the stable microbial growth in liquid culture, where the polysaccharide in question served as the sole source of carbon and energy. Turbidimetric analysis using cetyltrimethylammonium bromide was additionally used for quantitative assessment of HA degradation (Svobodová et al., 2025). High-performance anion-exchange chromatography with pulsed amperometric detection (HPAEC-PAD) was used to analyze the formation of oligohyaluronans according to a previously described procedure (La Cono et al., 2023). Briefly, culture medium supernatants (300 μL aliquots) were first mixed with absolute ethanol to a final ethanol concentration of 70% (vol/vol), centrifuged (10,000 *g*, 5 min), and filtered through 0.45-μm nylon filters (Cosela S. L.). Analysis of supernatants was performed at 30 °C using a Dionex ICS3000 HPAEC-PAD system with a CarboPack PA-100 anion exchange column (4 mm × 250 mm) coupled to a CarboPac PA-100 guard column (4 m × 50 mm) and an autosampler (model AS-HV) at a flow rate of 0.5 mL min^−1^, with a mobile phase contained 10 mM NaOH from start to finish, and two gradients with CH_3_COONa. The first step consisted of an 80-min gradient from 0 to 60 mM CH_3_COONa, and the second from 60 to 160 mM over 20 min. Finally, the column was equilibrated to initial conditions. The eluents were degassed with helium, and peaks were analyzed using Chromeleon software. Identification of certain reaction products was performed by comparison with standards (provided by Evonik, Essen, Germany), which include oligohyaluronans in the HA_2_-HA_70_ range.

For quantitative measurements of polysaccharide lyase activity, the isolates were grown in optimal conditions either on HA or CND as sole carbon and energy source until the end of exponential phase. Cells were separated from the supernatant by centrifugation for 30 min (15,000 g, 10 °C). The cells were resuspended in 20 mM phosphate buffer (pH 7.0), containing 2 M NaCl, 5 g l^−1^ KCl and 1 mM MgCl_2_, followed by sonication using SoniPrep 150plus (MSE) at 4 °C. Culture supernatants were concentrated 5–10 times using ultrafiltration modules Amicon 10 kDa (Merck). Thus, two fractions of cell lysate from cultures grown on HA and CND, respectively, and two fractions of concentrated culture supernatants were obtained for each of the strains and used for detection of polysaccharide lyase activity.

Reaction mixture (total volume 250 μL) contained 25 μL substrate (HA or CND at final concentration of 0.2 and 0.1%, respectively), 25 μL of cell lysate (CL) or culture broth (CB) and the remaining volume of 2M NaCl in phosphate buffer. After mixing in 0.5 mL tubes the samples were vortexed and stored at 10 °C until measuring. The lyase activity was monitored in UV-transparent 96-well microplates (UVMAX, SPL Life Science, Korea) at 30 °C by measuring increase in absorbance at *λ* = 235 nm continuously every 5–20 min using spectrophotometer SpectroStar Nano (BMG Labtech, Germany). Increase in A_235_ indicate formation of unsaturated oligomers of uronic acids (ΔUA). Parallel incubations of substrates (HA and CND) as well as fractions of cell lysates and culture broths served as negative controls. All measurements were performed in triplicates. Concentrations of ΔUA were calculated by using molar extinction coefficients: 5500 M^−1^ cm^−1^ and 5,260 M^−1^ cm^−1^ for unsaturated derivatives of HA (Kurata et al., 2015) and for CND (Williams et al., 2017), respectively. Protein concentrations were measured by Bradford method (Bradford, 1976). One unit of enzyme activity (U) was defined as 1 μmol of ΔUA formed per minute.

### Genomic sequencing and phylogenetic analysis

Genomic DNA isolation, DNA library preparation, sequencing as well as genome assembly were performed as described earlier (Sorokin et al., 2022; La Cono et al., 2020, 2023). Briefly, after sedimentation of biomass from 3 mL of grown isolate cultures H-hyl1, H-Hyl2 and H-hyl3 or SSP and VSALT enrichments by centrifugation (10,000 *g*, 5 min), total DNA was extracted using a GNOME DNA kit (MP biomedicals, Irvine, CA, USA) according to the manufacturer’s instructions. DNA was visualized on agarose gel (0.8% w/v). The quantity of DNA was additionally estimated with a QubitTM 4 Fluorometer (Thermo Fisher Scientific, Waltham, MA, USA), and send to Macrogen Europe BV for whole-genome shotgun sequencing using the Illumina ® NovaSeq 6,000 platform (San Diego, CA, United States) by Macrogen Europe BV using the 2 × 150 bp short insert paired-end library (Illumina Nextera DNA XT). The ligation sequencing kit SQK-NBD114.24 (Oxford Nanopore Technologies, UK) was used to prepare the DNA libraries according to the manufacturer’s protocols with slight modifications with the incubation time (the end-prep reaction time was increased to a total of 40 min). Long reads sequencing was performed using the MinION™ Oxford Nanopore Technologies platform (Oxford, UK). Hybrid assembly (short + long reads) for each sample was performed by Unicycler v. 0.4.9 program (Wick et al., 2017) using default parameters. Geneious Prime 2025.2.2 software^1^ was used to verify consistency and refine some contigs.

For genome-based phylogenetic reconstructions, 122 archaeal single-copy conservative marker genes were used as described previously (Parks et al., 2018; Rinke et al., 2021). The genome tree was performed using classify_wf workflow in GTDB-Tk (V1.7.0) based on Release 207 in Genome Taxonomy Database^2^ (Chaumeil et al., 2019). The trees were built using the IQ-TREE 2 program (Minh et al., 2020), with fast model selection via ModelFinder (Kalyaanamoorthy et al., 2017) and ultrafast approximation for phylogenetic bootstrap (Hoang et al., 2018) as well as approximate likelihood-ratio test for branches (Anisimova and Gascuel, 2006). The phylogenetic tree was polished using iTOL v.6.5.8 (Letunic and Bork, 2019).

### Genome analysis

The genomes of strains H-hyl1, Hhyl-2 and H-hyl6 were annotated with NCBI Prokaryotic Genome Annotation Pipeline (PGAP v. 6.10) (Tatusova et al., 2016). Identification of genomic islands (GI) was performed by IslandViewer 4 online tool (Bertelli et al., 2017). Whole-genome comparison of *Natronoarchaeum* and *Haloarcula* isolates was conducted by using three different methods: Average Nucleotide Identity (ANIb and ANIm), using JSpeciesWS web server; Average Amino acid Identity (AAI) by the AAI calculator online of Kostas lab^3^ and DDH by the Genome-to-Genome Distance Calculator 2.1 online tool^4^ (Richter et al., 2016; Rodriguez-R and Konstantinidis, 2014). Circular genomic map representation was performed by Circular-Plot tool inside Artemis v. 18.2.0 program (Carver et al., 2012). Extraction of genomic map figure was performed by Geneious Prime 2025.2.2 software. Carbohydrate-active enzymes (CAZymes), namely glycoside hydrolases and polysaccharide lyases of the GH and PL families, respectively were first screened in the genomes using dbCAN3 (Drula et al., 2022; Zheng et al., 2023) and then verified by using NCBI blastp program (Altschul et al., 1997) against Swiss-Prot database (Boutet et al., 2016), UniProt Blast and HHMER_pfam v3.3 Mistry et al., 2013) for domain architecture with default thresholds. Enzyme localization was predicted using SignalP v.6.0 (Teufel et al., 2022).

### Data deposition, gene bank accession numbers and data availability statement

All data used and analyzed in this study have been included in the present article and its Supplementary Files. All whole genome and metagenome sequencing information were deposited in NCBI GenBank database and are freely available through the NCBI data under the corresponding BioProject, BioSample, and accession numbers. The assembled genome sequence of *Natronoarchaeum rubrum* H-hyl1 was submitted to NCBI (BioProject PRJNA1332976; BioSample SAMN51785343). This Whole Genome Shotgun project has been deposited at DDBJ/ENA/GenBank under the accession JBRKBG000000000. The version described in this paperis version JBRKBG010000000. The assembled genome sequence of *Haloarcula* sp. H-hyl2 was submitted to NCBI (BioProjectPRJNA1333890; BioSample SAMN51818556). This Whole Genome Shotgun project has been deposited at DDBJ/ENA/GenBank under the accession JBRIIJ000000000. The version described in this paper is version JBRIIJ010000000. The assembled genome sequence of *Haloarcula* sp. H-hyl6 was submitted to NCBI (BioProject PRJNA1333891; BioSample SAMN51818726). This Whole Genome Shotgun project has been deposited at DDBJ/ENA/GenBank under the accession JBRIIK000000000. The version described in this paperis version JBRIIK010000000. The metagenome sequencing data of the hyaluronate-degrading enrichment VSALT was submitted to NCBI (BioProject PRJNA1406516; BioSample SAMN54773696). This Whole Genome Shotgun project has been deposited at DDBJ/ENA/GenBank under the accession JBUBAT000000000. The version described in this paper is version JBUBAT010000000. The metagenome sequencing data of the hyaluronate-degrading enrichment SSP was submitted to NCBI (BioProject PRJNA1406516; BioSample SAMN54773624). This Whole Genome Shotgun project has been deposited at DDBJ/ENA/GenBank under the accession JBUBAS000000000. The version described in this paper is version JBUBAS010000000. All statements regarding data availability, finding resources and conflict of interest disclosure have been provided.

## Results and discussion

### Enrichment, isolation and cultivation of HA and CND-utilizing haloarchaea

All primary enrichments obtained from collected material (brine, sediments, or freshly harvested salt) from five hypersaline lakes and salterns located across Eurasia (from Portugal to Vietnam) (Supplementary Figure S1) were assessed as positive for HA growth and degradation within three months of cultivation. This was confirmed by the increased turbidity (measured as increase in optical density at OD 600 nm) accompanied by an intensive pink-red coloration. All were further converted to a sediment-free stage after three to five successive subcultures at a ratio of 1:100 ol/vol) on the same liquid mineral medium supplemented with either 2MDa or 50 KDa HA (0.05% w/vol) and a mixture of bacteria-specific antibiotics (streptomycin and vancomycin, 100 mg l^−1^ each). After a secondary selection of the fastest-growing enrichments, those originated from hypersaline lakes of Kulunda (Russia), as well as the marine solar salterns of Trapani (Italy) and Nha Trang (Vietnam) were chosen for further isolation of hyaluronate-utilizing haloarchaea. These cultures were subjected to serial antibiotic-free dilution-to-extinction steps and typically yielded positive results (colony formation) up to 10^−8^–10^−9^. Final purification was achieved by selecting individual colonies on solid media, which were tested for their ability to grow on HA by transferring them back to liquid medium and simultaneously measuring both their growth and hyaluronate degradation. It should be noted that the described strategy scored as 100% positive in obtaining of hyaluronan-degrading enrichments from all samples used through this study. Noteworthy, further isolation yielded only one site-specific axenic culture of HA-utilizing haloarchaea in each of three enrichments, named H-hyl1 (Kulunda), H-hyl2 (Trapani) and H-hyl6 (Nha Trang), respectively. Moreover, the last two isolates were very similar in appearance, growth dynamics and, as we later found out, they belonged to the same genus. This indirectly indicates that a novel haloarchaeal metabolism based on GAG degradation is widespread in hypersaline habitats worldwide, albeit represented by a relatively low number of specialists. The remaining positive enrichments, SSP (Samouco solar salterns, Alcochete, Portugal) and VSALT (freshly harvested salt in the Cam Ranh region, Vietnam), were not discarded from the study but were used for subsequent genomic/biomolecular studies. Their detailed characterization revealed the presence of haloarchaea genomically similar to isolate H-hyl1.

**Figure 1:**
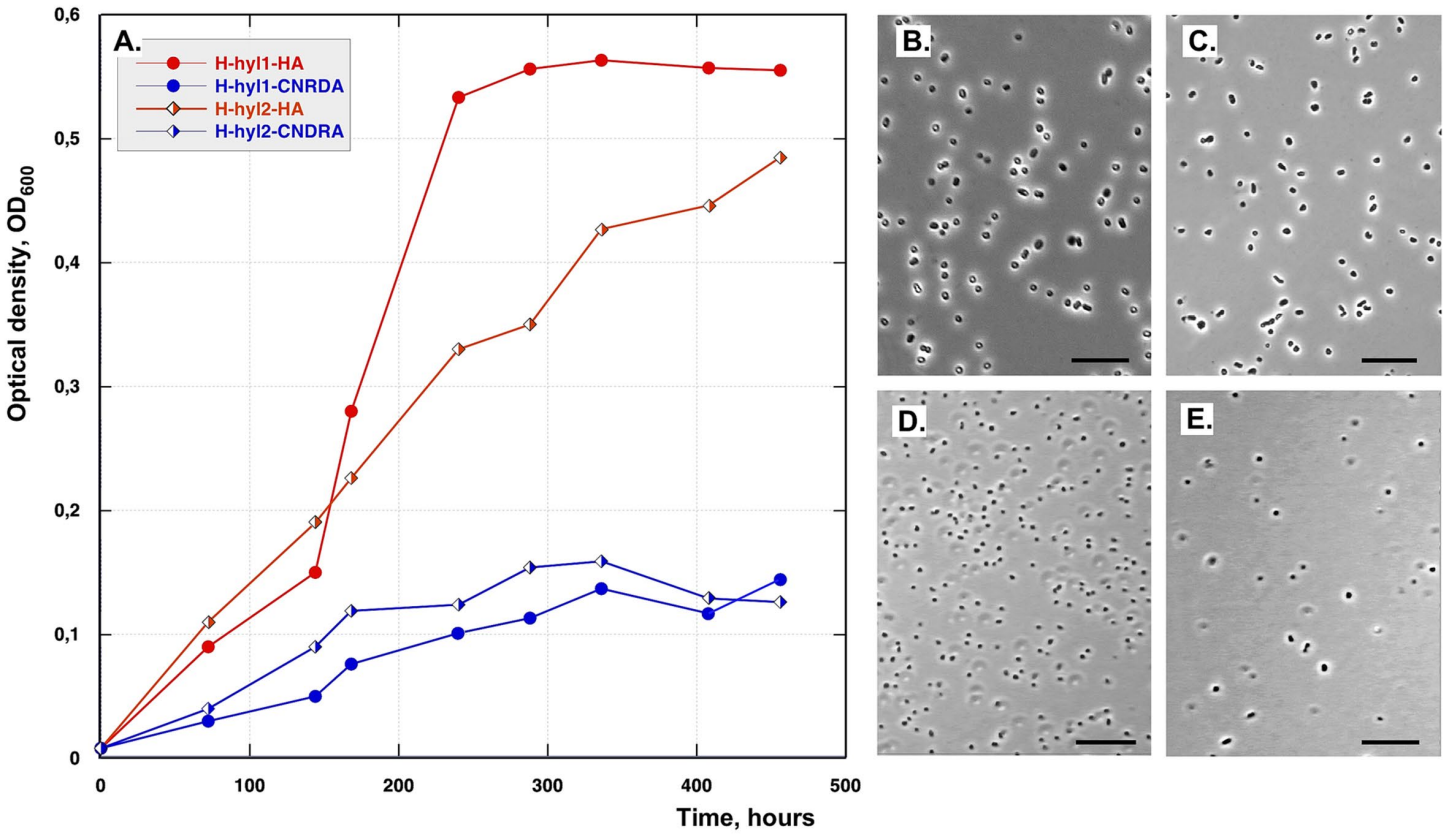
Growth of pure cultures of *H*-hyl1 and *H*-hyl2 on 50 kDa sodium hyaluronate (Evonik) and chondroitin sulfate A (Sigma-Aldrich C9819) **(A)**. Both GAGs were added as the sole carbon and energy source (initial concentration 0.05% [w/vol]). The OD_600_ values presented in the graph are the average and are based on three replicates of cultivation. Cell morphology of the GAG-depolymerizing haloarchaea *H*-hyl1 **(B,C)** and *H*-hyl2 **(D,E)** grown on hyaluronate HA50 (Evonik) and chondroitin sulfate A, respectively. The scale bar corresponds to 10 μm.

Cells of hyaluronan-utilizing H-hyl1, H-hyl2 and H-hyl6 were predominantly non-motile flat, irregular coccoids or short rods, ~1-2 μm in diameter, and frequently containing light-refracting inclusions that stained positively with Nile-Blue for polyhydroxyalkanoates (the synthesis of which was further confirmed by genome analysis). All isolates demonstrated relatively rapid growth on hyaluronate with variable polymerization degree, from 2 MDa to 50 KDa, and were also capable of utilizing chondroitin sulfate A (CND) as a sole source of carbon and energy, albeit at a significantly slower rate (Figure 1). From the tree isolates, H-hyl6 was the best-fit for using the CND. CND is a sulfated GAG composed of an alternating chain of *N*-acetyl-*D*-galactosamine and glucuronic acid dimers, in which the sulfate group is esterified at the 4-position of the amino sugar residue. When using other known forms of chondroitin (chondroitin B [dermatan sulfate], C, D and E), which differ in the position and number of sulfate groups, growth on them was very weak if any in all the isolates obtained. Overall, all HA-utilizing isolates possess a very narrow range of polysaccharide growth substrates, limited in addition to the aforementioned GAGs (hyaluronate and chondroitin sulfate A) to only starch, dextran, glycogen and, in addition, H-hyl1 was also able to grow with laminarin. The following polysaccharides were tested negative as growth substrates: agarose, alginate, amorphous cellulose and chitin, beech xylan, *β*-mannanans, alginate, fucoidan, heparin and keratan sulfate, pectin (apple or citrus), polygalacturonate, and rhamnogalacturonan.

After 20 days of cultivation at 40 °C in the liquid mineral medium supplemented with HA (0.05% w/vol), the supernatant of H-hyl1, H-hyl2, and H-hyl6 cultures was used for a detailed analysis of polysaccharide degradation. In all cultures, the added 50 kDa sodium hyaluronate was completely degraded, and numerous hyaluronan oligomers were detected using high-performance anion exchange chromatography (Supplementary Figure S2). It should be noted that, although oligo-hyaluronans in the HA_6_-HA_40_ range were found in all samples, the HA_2_ disaccharide was potentially identified only in H-hyl6 culture and only in insignificant quantities. As will be shown in the subsequent section devoted to the analysis of genomes, it is this dimer that is transported into the cells of HA-utilizing haloarchaea for subsequent hydrolysis to form monosaccharides, and its absence in the supernatant indicates an active process of its uptake.

**Figure 2:**
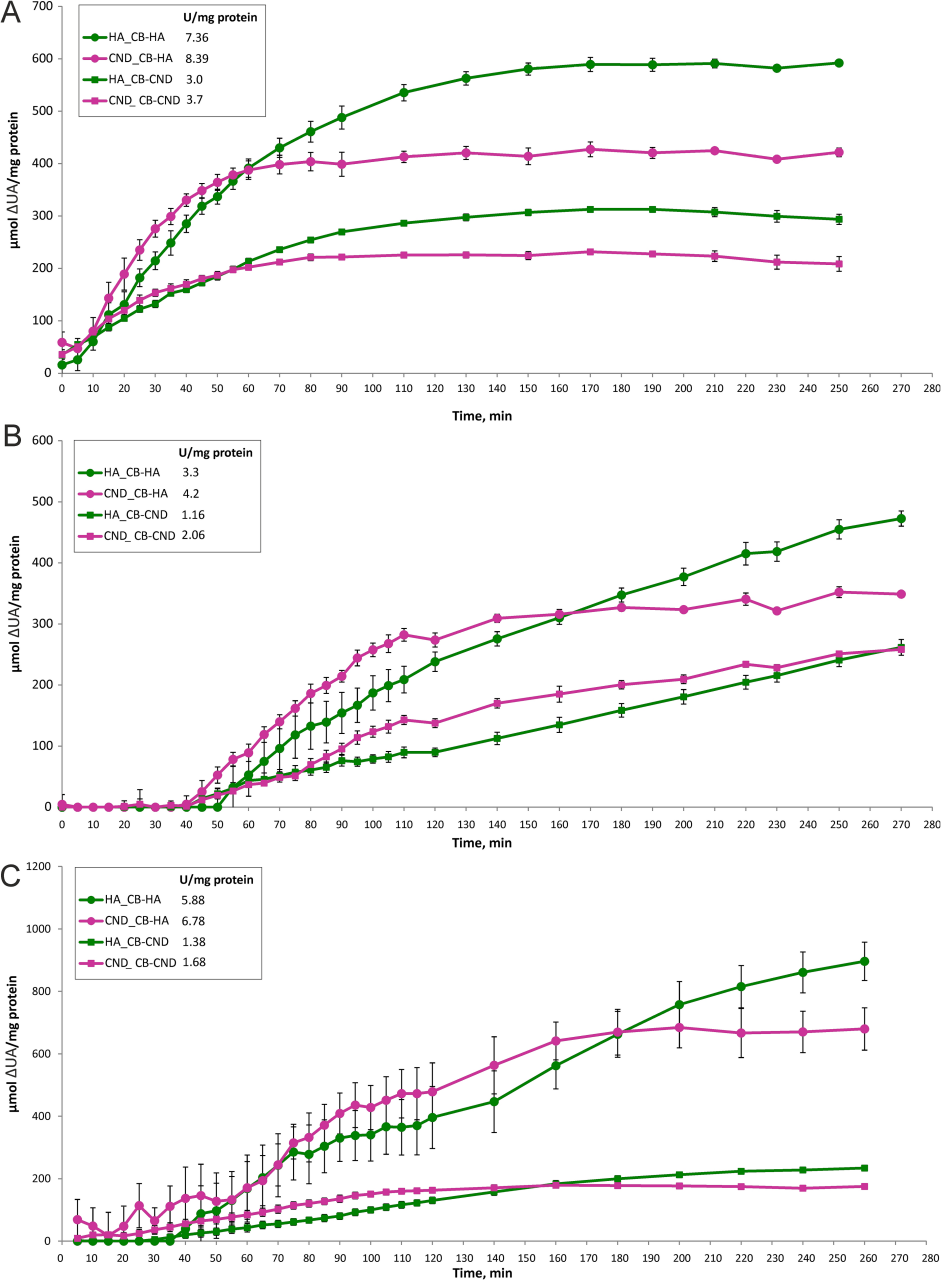
Polysaccharide lyase activities curves of culture broths (CB) of three haloarchaeal strains: **(A)** CB of H-hyl1, grown on hyaluronate (CB-HA) and chondroitin sulfate (CB-CND); **(B)** CB of H-hyl2, grown on hyaluronate (CB-HA) and chondroitin sulfate (CB-CND); **(C)** CB of H-hyl6, grown on hyaluronate (CB-HA) and chondroitin sulfate (CB-CND). Enzymatic activities towards HA signed by green lines, activities towards CND signed by magenta lines. Specific activities (U/mg protein) are shown in boxes for each tested CB fraction.

Formation of unsaturated residues from both substrates (HA and CND) was detected for all fractions of culture supernatants of all three strains, indicating the presence of extracellular polysaccharide lyase(s) (Figure 2). At the same time, no activities were detected in the cell lysates demonstrating that the excreted polysaccharide lyases are not associated with the cell surface. The cell-free lyases in all tested haloarchaea were active against both HA and CND. However, cultures grown on HA, possessed hyaluronidase and chondroitinase activities 2–3 times higher than those grown on CND. This might be an indication that all isolates use a single hyaluronate lyase for initial depolymerization of both GAGs. In H-hyl1 the activity was detected nearly without a lag phase with the high rate being maintained until 50–60 min of reaction time, after which a slowdown and a plateau were reached (Figure 2A). In the other two strains, there was a substantial delay for the activity offset showed activity starting from 30 to 50 min of incubation and the specific lyase activities for both polymers were lower than in H-hyl1 (Figures 2B,C).

### Phylogenetic placement of the isolates

**Figure 3:**
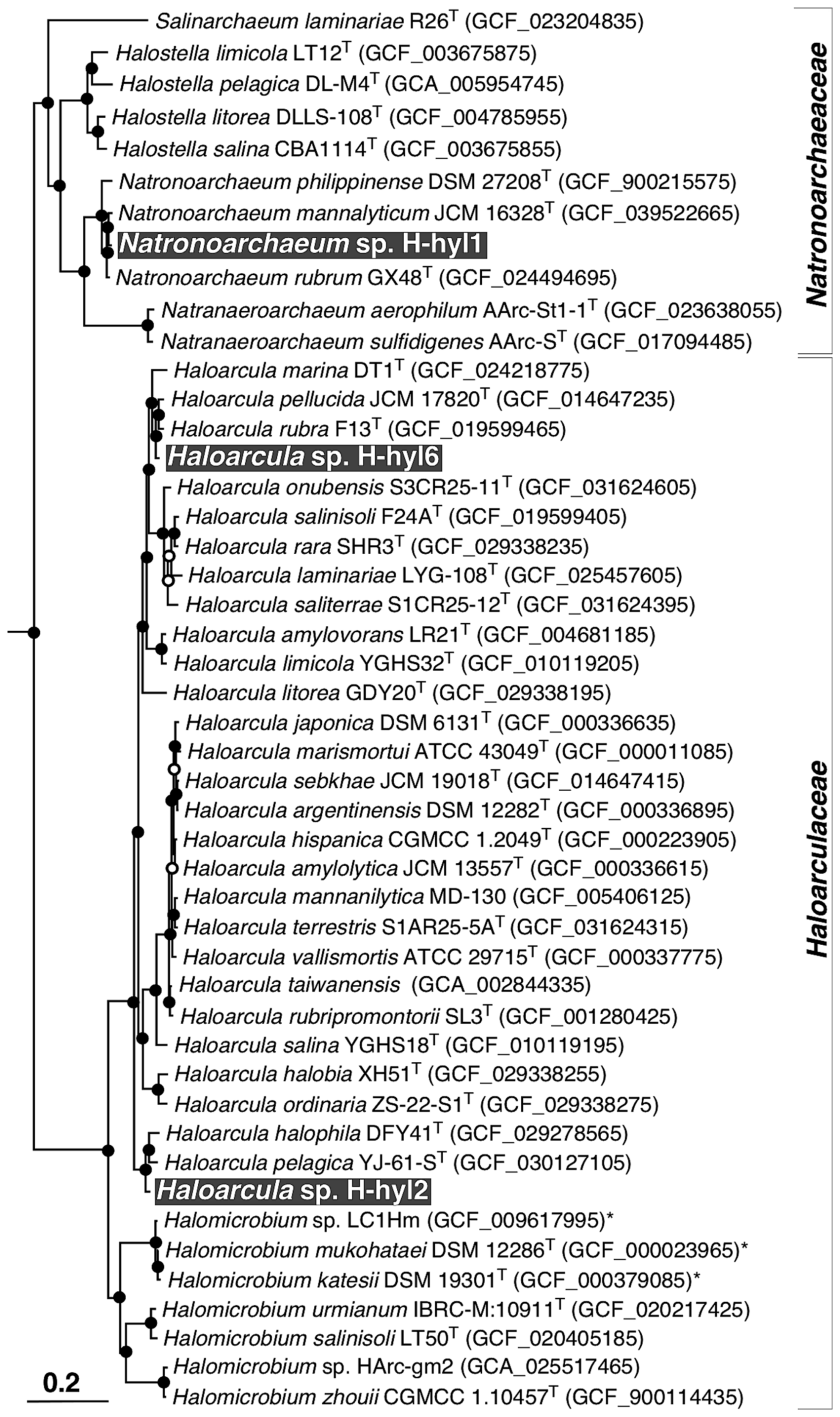
A maximum likelihood phylogenetic tree based on 122 conserved protein markers (Parks et al., 2018) demonstrating the positions of three GAG-utilizing haloarchaea (in bold) within the families *Natronoarchaeacea*e and *Haloarculaceae*, respectively. Taxonomic designations correspond to the Genome Taxonomy Data Base (GTDB). Trees were constructed using IQ-TREE 2 with the approximate likelihood ratio test for branches. Branch lengths correspond to the number of substitutions per site with corrections associated with the models. Black and open circles at nodes indicate that the percentage of corresponding support values was >70 and >50, respectively.

Genome sequencing and detailed phylogenomic analyses revealed that all GAG-utilizing isolates, despite the possession of a novel metabolic pathway, belong to well-characterized haloarchaeal genera. The genome of H-hyl1 (JBRKBG010000000) contains two rRNA operons with identical 16S rRNA genes, closely related to representatives of the genus *Natronoarchaeum*, *Nac. mannanilyticum* and *Nac. rubrum* (99.12 and 98.98% 16S rRNA gene sequence identity, respectively). The H-hyl2 genome (JBRIIJ010000000) harbors two rRNA operons, with highly dissimilar *rrn*A and *rrn*B genes (<93.29% gene identity), both of which are distantly related to three members of the genus *Haloarcula*, *Har. pelagica*, *Har. halophila* and *Har. montana* (<98.09% of 16S rRNA gene sequence identity). Finally, the H-hyl6 genome (JBRIIK010000000) contains three rRNA operons, with two identical *rrn*B genes and one highly divergent *rrn*A gene (<94.23% gene identity). Like the H-hyl2 isolate, it also belongs to the genus *Haloarcula*, although depending on the 16S rRNA genes analyzed, it is more closely related to different species of this genus. In particular, the *rrn*A gene of strain H-hyl6 is more closely related to *Har. rubra* and *Har. pellucida* (97.49 and 97.22% gene sequence identity, respectively), whereas its *rrn*B genes are more closely related to *Har. marina* and *Har. limicola* (97.42 and 97.21% of 16S rRNA gene sequence identity, respectively). In any cases, analysis of these intraspecific polymorphic 16S rRNA gene sequences in H-hyl2 and H-hyl6 isolates is consistent with the phylogenetic position of these strains as novel species-level lineages within the genus *Haloarcula*. The maximum likelihood phylogenomic tree constructed from the comparison of 122 conserved single-copy protein markers supported the assignment of strain H-hyl1 to the genus *Natronoarchaeum,* and the strains H-hyl2 and H-hyl6 to the genus *Haloarcula* (Figure 3). The whole genome comparison showed that the new isolates differ from validly described closest related species, demonstrating AAI and ANI values below 90% between H-hyl1 and *Natronoarchaeum* species and between H-hyl2, H-hyl6 and *Haloarcula* species (Supplementary Tables S1–S3). The same low values of identity were obtained when comparing H-hyl2 and H-hyl6 with each other (AAI < 74% and ANI < 86%). All values are well below the proposed interspecies cutoff values (Konstantinidis and Tiedje, 2005; Rodriguez-R and Konstantinidis, 2014), confirming that all these H-hyl isolates represent three distinct species within genera *Natronoarchaeum* and *Haloarcula*.

### Functional genome analyses of GAG-utilizing isolates

The genome of *Natronoarchaeum* sp. H-hyl1 was composed of a circular chromosome of 3,780,195 bp with 3,803 CDS, 49 tRNA, 2 operons rRNA (16S-23S-5S), and three circular plasmids of 302,444 bp, 133,220 bp, and 78,115 bp, respectively. The larger plasmid (pH-hyl1-01) also harbours an rRNA operon (16S-23S-5S), while a PL8 gene was identified in the plasmid pH-hyl1-02. The genome of *Haloarcula* sp. H-hyl2 was composed of 16 contigs, with contig 3 and 5 being circular plasmids (429,535 bp and 169,363 bp respectively). Overall, they include 4,410 CDS, 2 rRNA operons, and 45 tRNA. A PL8 gene was identified in the plasmid 2 (contig 5). The genome of *Haloarcula* sp. H-hyl6 was composed of 5 contigs, with contig 3 and 5 being circular plasmids (476,033 bp and 289,757 bp respectively). Overall, they consist of 4,672 CDS, 3 rRNA operons, and 46 tRNA. A PL8 gene was identified in the plasmid 2 (contig 5) (Table 1).

**Table 1:** General information for H-hyl1, H-hyl2 and H-hyl6 genomes.

Attribute	*Natronoarchaeum* sp. H-hyl1	*Haloarcula* sp. H-hyl2	*Haloarcula* sp. H-hyl6
Genome composition	1 circ. Chromosome, 3 circ. Plasmids	14 contigs + 2 circ. Plasmids	3 contigs + 2 circ. Plasmids
Genome size	3,780,195 bp (chr) 302,444 bp (pl1) 133,220 bp (pl2) 78,115 bp (pl3)	3,794,954 bp 429,535 bp (pl1) 169,363 bp (pl2)	3,899,493 bp 476,033 bp (pl1) 289,757 bp (pl2)
GC content	65.2%	64.1%	64.6%
DNA coding region (%)	3,678,418 bp (85.7%)	3,895,093 bp (88.6%)	4,095,784 bp (87.8%)
Total genes	4,323	4,463	4,731
tRNA genes	50	45	46
rRNA genes (5S-16S-23S)	9	6	9
ncRNA genes	2	2	2
CRISPR repeat region	–	2	2
Protein-coding genes	4,262	4,410	4,674
Average gene length	850.9 bp	872.8 bp	865.7 bp
Max gene length	8,772 bp	5,568 bp	8,427 bp

In line with the relatively limited catabolic potential towards the various polysaccharides tested, the genomes of H-hyl isolates contain a rather poor set of genes encoding carbohydrate active enzyme (CAZymes) involved in polysaccharide depolymerization; namely, glycoside hydrolases (GH), poly(oligo)saccharide/carbohydrate esterases (CE) and polysaccharide lyases (PL) (Table 2). Strains H-hyl1, H-hyl2, and H-hyl6 grew on *α*-glucans, likely with the help of ten, twelve, and six GH13 family amylose endo-α-1,4-glucosidases, and four, five, and three GH15 family glucoamylases, respectively. Remarkably, only one CAZyme, encoded in all three genomes, can be classified as a determinant minimally required for hyaluronan degradation. In all strains, this enzyme, a member of the polysaccharide lyase family 8 (PL8) was encoded on one of their plasmids (a more detailed analysis of both plasmids and PL-containing loci is provided below). The H-hyl lyase open reading frames (locus tags Hhyl1_21205, Hhyl2_20155 and Hhyl6_23175) encode deduced proteins consisting of 823, 804, and 805 amino acid residues and having theoretical *pI* values of 4.38, 4.64, and 4.56, respectively. Bioinformatic analysis using SignalP 6.0 program^5^ revealed the presence of predicted Tat/SPI signal peptide (signal peptides transported by the twin-arginine translocation [Tat] pathway) and cleaved by signal peptidase SpI between positions 48/49, 30/31, and 29/30, respectively. The absence of any additional transmembrane anchors strongly suggests that all H-hyl lyases are extracellular.

**Table 2:** Carbohydrate active enzyme (CAZymes) found in the *Natronoarchaeum* sp. H-hyl1, *Haloarcula* sp. H-hyl2 and *Haloarcula* sp. H-hyl6 genomes: glycosyl hydrolases (GH), carbohydrate esterases (CE) and polysaccharide lyases (PL).

CAZy family	Putative function	Organism		
		H-hyl1	H-hyl2	H-hyl6
GH0	Endo-1,4- β-mannosidase, Cpl		1	1
GH2	β-galactosidase/*β*-glucuronidase, Cpl	3		
GH2	β-galactosidase/β-glucuronidase, Extra (Tat/SPI)	1		
GH3	β-glucosidase, Cpl	2		
GH3	β-glucosidase, Extra (Tat/SPI)	1		
GH4	α-galactosidase, Cpl		1	1
GH5_13	endo-1,4-beta-mannosidase, Cpl	1	1	
GH5_13	endo-1,4-beta-mannosidase, Extra (Tat/SPI)			1
GH13_2	amylose endo-α-1,4-glucosidase, Extra (Tat/SPI)	1		
GH13_4	sucrose α-1,2-glucosidase, Cpl		1	
GH13_16	trehalose synthase / maltose glucosylmutase, Cpl		2	1
GH13_31	amylose endo-α-1,4-glucosidase, Cpl	2	5	3
GH13_43	amylose endo-α-1,4-glucosidase, Cpl		1	
GH13_49	amylose endo-α-1,4-glucosidase, Cpl	6	2	1
GH13_49	amylose endo-α-1,4-glucosidase, Extra (Tat/SPII)	1	1	1
GH15	glucan 1,4-α-glucosidase, Cpl	4	4	2
GH15	glucan 1,4-α-glucosidase, Extra (Tat/SPI)		1	1
GH31_3	putative α-glucosidase, Cpl		1	
GH32	sucrose β-2,1-fructosidase, Cpl	3	2	2
GH36	α-amylase, Cpl		1	
GH37	trehalose α-1,1-glucosidase, Cpl	1	1	
GH42	β-galactosidase, Cpl		1	1
GH63	α-glucosidase; trehalase, Cpl		1	1
GH65	maltose phosphorylase, Cpl	1		
GH68_2	sucrose β-2,1-fructosidase, Cpl	1	1	1
GH77	4-α-glucanotransferase, Cpl	1	2	
GH78	α-L-rhamnosidase, Cpl	4		
GH81	glucan endo-β-1,3-glucosidase, Cpl			1
GH81	glucan endo-β-1,3-glucosidase, Extra (Tat/SPI)		1	
GH88	unsaturated-β-1,3-glucuronidase, Cpl	1	2	2
GH97	amylose exo-α-1,4-glucosidase, Cpl			1
GH97	amylose exo-α-1,4-glucosidase, Extra (Tat/SPI)	1	2	3
GH99	N-glycan endo-α-1,2-mannosidase, Extra (Tat/SPII)	1		
GH106	α-L-rhamnosidase, Cpl	1		
GH154	exo-β-1,6-glucuronidase, Cpl	2	1	1
GH176	starch endo-α-1,6-glucosidase, Cpl		2	2
CE4	Poly-oligosaccharide deacetylase, Cpl	4	2	4
CE4	Poly-oligosaccharide deacetylase, Extra (Sec/SPII)	1		
CE4	Poly-oligosaccharide deacetylase, Extra (Tat/SPI)			1
CE unclassified	ChbG/HpnK family carbohydrate deacetylase, Cpl			1
PL8	Glycosaminoglycan (GAG) polysaccharide lyase, Extra (Tat/PSI)	1	1	1
	Total number of CAZymes	45	47	37

Cpl, cytoplasmic location of enzyme; Extra, extracellular location of enzyme with type of signal peptide in parenthesis.

According to the CAZy classification database (Lombard et al., 2014), the polysaccharide lyase family 8 currently includes four subfamilies of secreted bacterial lyases (chondroitin AC and ABC lyases, hyaluronate lyase and xanthan lyase), capable of cleaving acidic polysaccharides such as xanthan gum and GAGs. In the latter type of polysaccharides, these PL8 enzymes, classified as GAG_lyases, recognize uronyl residues containing 1,4-*β*-D-hexosaminyl and 1,3-β-D-glucuronosyl or 1,3-α-L-iduronosyl linkages and cleave their glycosidic bonds via a β-elimination reaction, forming a double bond between C-4 and C-5 of the non-reducing terminal uronyl residues of the released disaccharides (Li and Jedrzejas, 2001; Stern and Jedrzejas, 2006; Jedrzejas, 2007). Thus, given that all H-hyl isolates obtained possess only one GAG_lyase, it is quite clear that this enzyme is responsible for the ability of the strains to grow also on chondroitin-4-sulfate which is also follows from the results of lyase activity tests described above. Apropos, a similar property has been demonstrated for various, though not all, cultured bacteria belonging to the genera *Bacillus*, *Streptococcus*, and *Vibrio*, which possess a single GAG_lyase and are capable of degrading both HA and chondroitin sulfate (Kurata et al., 2015; Wang et al., 2021).

**Figure 4:**
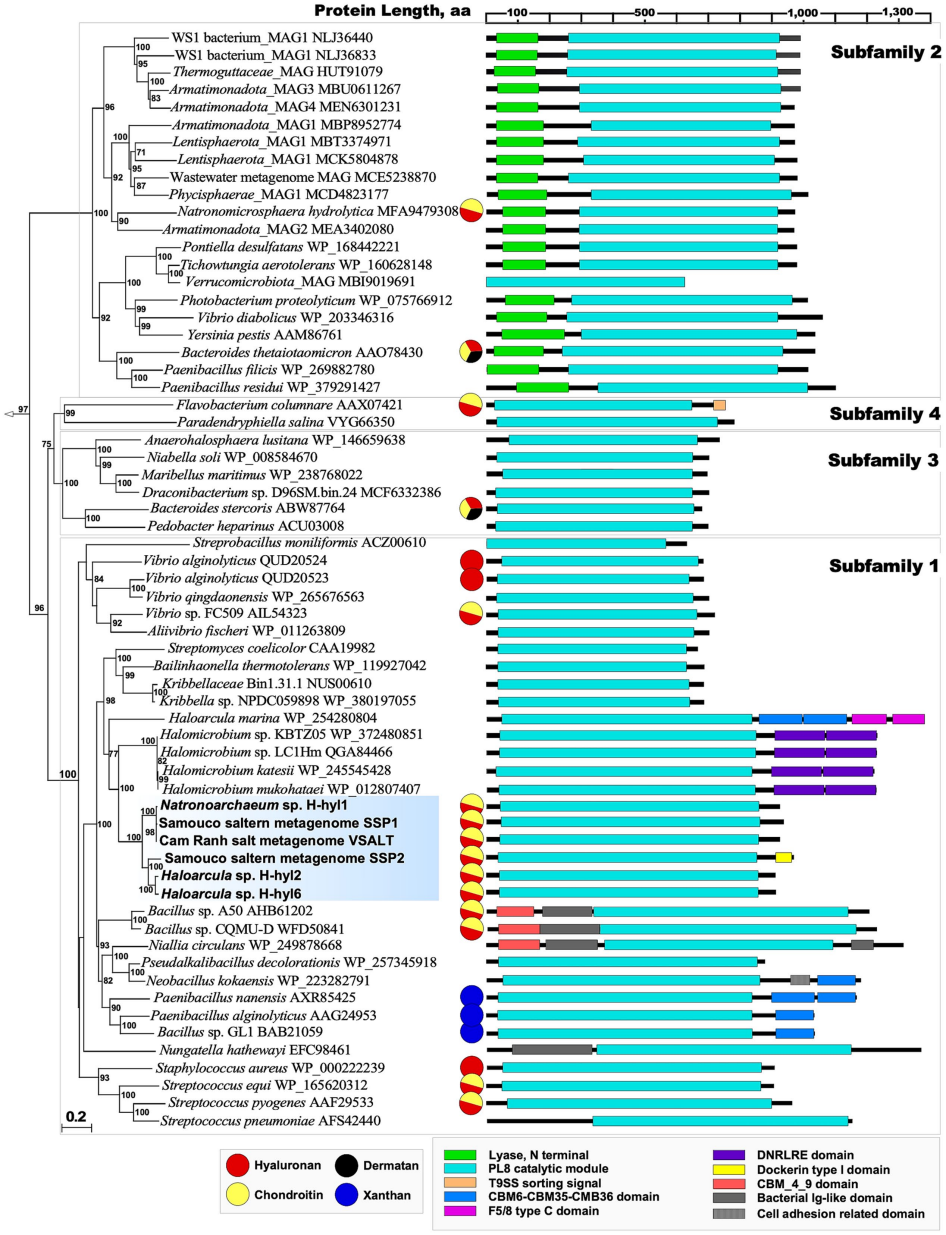
Maximum likelihood phylogenetic tree placing haloarchaeal hyaluronate/chondroitin lyases among other GAG- and heteropolysaccharide-degrading enzymes of the PL8 family. The phylogenetic tree was generated using MEGA-X (Kumar et al., 2018) and the tree with the highest log-likelihood is shown. Totally 63 sequences belonging to all known PL8 subfamilies were retrieved from GenBank and only catalytic lyase modules were considered in the phylogenetic analysis. Hyaluronate/chondroitin lyase from *Hhyl* isolates and SSP and VSALT enrichments are highlighted in bold. Bootstrap values (1,000 replicates) are shown next to the branches. The tree was rooted with *Bacteroidetes intestinalis* heparinase of the PL13 family (WP_394798508). The bar represents 0.2 amino acid substitutions per site. The architectural structure and length of the corresponding fragments of PL8 polysaccharide lyases are presented based on protein domains identified using InterPro prediction (Mitchell et al., 2019) and NCBI conserved domain search (Marchler-Bauer et al., 2017; Wang et al., 2023). The catalytic domains of PL8 polysaccharide lyase are shown in light blue, while the identity of all other domains is indicated by different colors in the figure. Domain abbreviations are as follows: CBM, carbohydrate binding module; T9SS, type IX secretion system sorting signals; F5/8 type C, blood coagulation factors V and VIII [discoidin] domain; Ig-like, immunoglobulin-like domain; DNRLRE, a protein domain, named after its characteristic DNRLRE sequence motif. The PL8 lyases from bacteria and haloarchaea described in this study, which have been experimentally shown to have the ability to grow and degrade either xanthan or various GAGs (hyaluronate, chondroitin, and dermatan), are indicated by circles located in front of the PL8 architectural structure with segments of the corresponding color.

To our knowledge, no archaeal prototypes of active GAG_lyases have been described and characterized prior to our study. Therefore, it is not surprising that a BLAST search of similar enzymes in the NCBI database using lyase catalytic modules revealed that these enzymes share some similarity to only five archaeal CAZymes. These were uncharacterized proteins containing the CBM96 carbohydrate-binding module, and putative PL8 lyases found in the genomes of four *Halomicrobium* species, including the chitinolytic strain LC1Hm WP_255318051 (La Cono et al., 2020) and the PL8 super-sandwich domain-containing protein WP_254280804 found in the genome of *Haloarcula marina* YSSS71 (Figure 4). However, even this clustering does not guarantee that these proteins are true hyaluronate lyases. Indeed, we attempted to grow the LC1Hm strain on both hyaluronate and chondroitin, but without success. The PL8 lyases found in *Halomicrobium* spp. and *Har. marina* YSSS71 most likely specialize in the degradation of other polysaccharides and differ from HA lyases. This is supported by their much more complicated architecture, due to the presence of various repeating C-terminal structures consisting of either CBM6/35/36, DNRLRE, or coagulation factor 5/8 domains (Figure 4). Nevertheless, despite these differences, the catalytic modules of archaeal PL8 lyases form a close group, distantly related (<45% amino acid sequence identity) to PL8 subfamily 1 lyases found in various high GC Gram-positive bacteria from the *Bacillales* order. This distant position, along with the minimal number of similar enzymes found in archaea, suggests that they were most likely acquired by horizontal gene transfer events from as-yet-unknown actinomycetes and possibly belong to a new branch of the PL8 subfamily 1.

**Figure 5:**
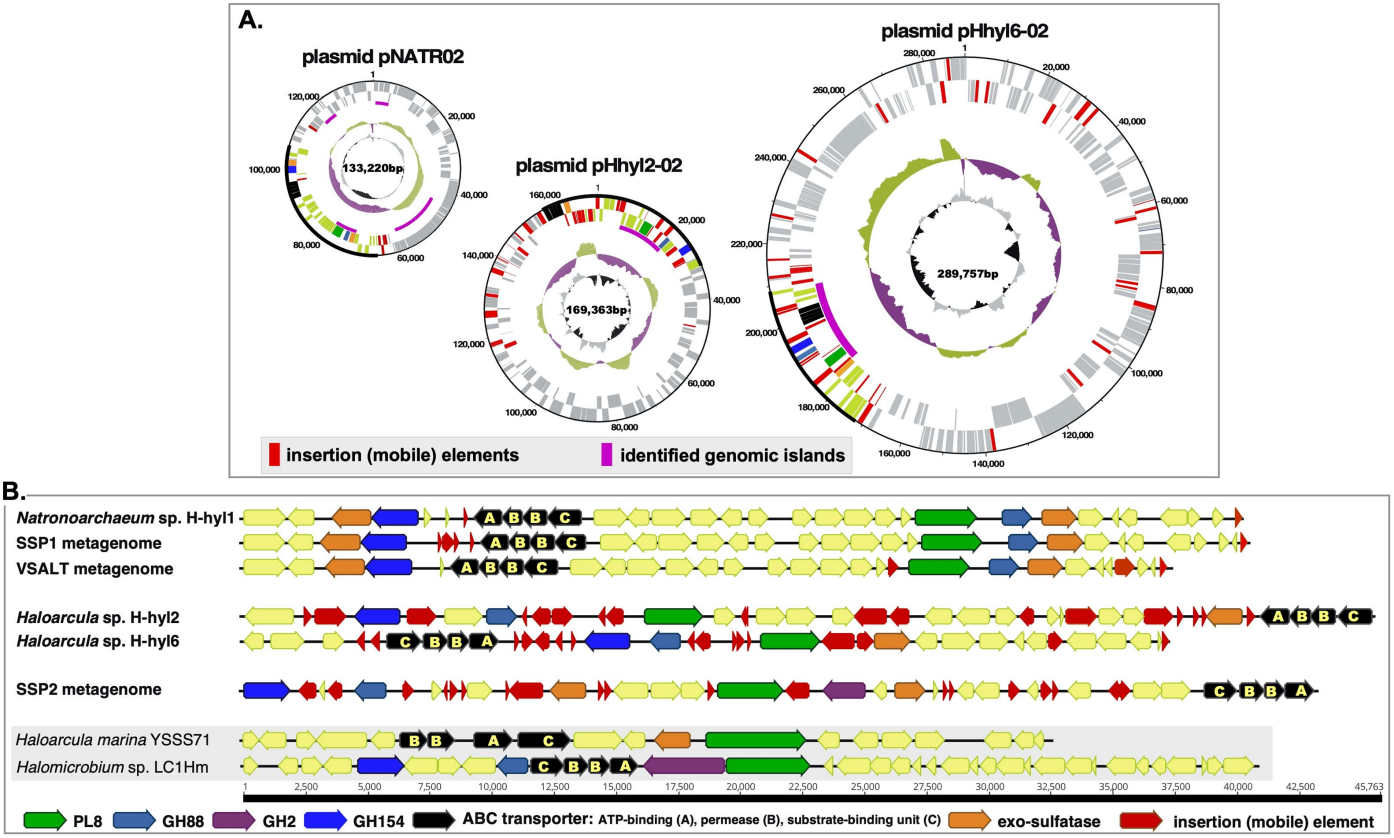
Graphical circular map of plasmids H-hyl1, H-hyl2, H-hyl6 in which GAG_lyase (PL8) gene was identified **(A)**. Rings from outside to center: (i) Polysaccharide utilization (PUL)-like locus shown as thick black segment (ii) forward strand genes, (iii) reverse strand genes (PUL-attributed genes and insertion elements are colored the same as in **B**); (iv) identified genomic island region (in purple); (v) GC content; (vi) GC skew (see methods). The genomic environment around the GAG_lyase gene (green) of plasmids H-hyl1, H-hyl2, H-hyl6 and metagenomic contigs found in the SSP and VSAL metagenomes **(B)**, with PUL-attributed genes highlighted (GH88 in azure, GH2 in purple, GH154 in blue, ABC oligosaccharide transporter system in black, and exosulfatase in orange). The genomic environment near the PL8 lyase genes found in *Haloarcula marina* YSSS1 plasmid3 (NZ_CP100410.1) and the *Halomicrobium* sp. LC1Hm plasmid pLC1Hm-01 (NZ_CP044130.1) is shown in the gray insert below for comparison.

Further indirect confirmations of the horizontal acquisition of GAG_lyase by H-hyl isolates are provided by analysis of its genomic environment, which revealed several noteworthy features. First, in all H-hyl strains, these lyases were encoded on the plasmids, not on chromosomes, and exclusively within genomic islands predicted by IslandViewer 4 (Bertelli et al., 2017). It should be noted that, although the lyase-containing plasmid pNATR02 of *Natronoarchaeum* sp. H-hyl1 harbors four identified islands, single genomic island was found in each of the lyase-containing plasmids pHhyl2-02 and pHhyl6-02 of *Haloarcula* strains (Figure 5). As mentioned above, GAG metabolism in archaea was unknown prior to our study. Therefore, to identify the minimal set of key enzymes required for the cleavage of these polymers and the uptake of the resulting oligosaccharides, we relied on well-characterized GAG degradation pathways in *Bacteroides* strains (Alvarez et al., 2025; Hameleers et al., 2024). Using this information, a similar enzyme repertoire was identified in haloarchaea. In addition to GAG_lyase, it includes a sulfatase for the formation of unsulfated chondroitin, exo-*β*-uronyl hydrolase from the glycoside hydrolase family GH88, and β-D-glucuronate dehydratase from the glycoside hydrolase family GH154 (Alvarez et al., 2025; Hameleers et al., 2024). Without exception, both GAG_lyase and GH88 are colocated in genomic islands predicted in H-hyl plasmids. Moreover, all other genes, constituting the minimal GAG depolymerization machinery are also located nearby in the same plasmids, namely in regions up to 46 kb long that are somewhat reminiscent of the polysaccharide utilization loci (PULs), described in various prokaryotes and catalogued in the open-source dbCAN-PUL database (Ausland et al., 2021).^6^ The “somewhat reminiscent” means to indicate that, along with the genes encoding key enzymes involved in glycosaminoglycan degradation and oligosaccharide transport, other genes encoding numerous non-CAZymes, hypothetical proteins and mobile elements are also present in the loci (Figure 5). Metagenomic analysis of hyaluronan-degrading SSP (Samouco, Portugal) and VSALT (Cam Ranh, Vietnam) enrichments revealed in both cases the presence of minimal GAG depolymerization PUL domains, structurally very similar to the PUL domain of H-hyl1 (Figure 1). Remarkably, the metagenome of SSP enrichment contains an additional PUL domain, completely distinct from all of our described domains (see below). We are currently attempting to isolate the host of this new PUL domain into pure culture.

### Genomic analysis and deduction of depolymerization of HA and CND by Hhyl isolates

**Figure 6:**
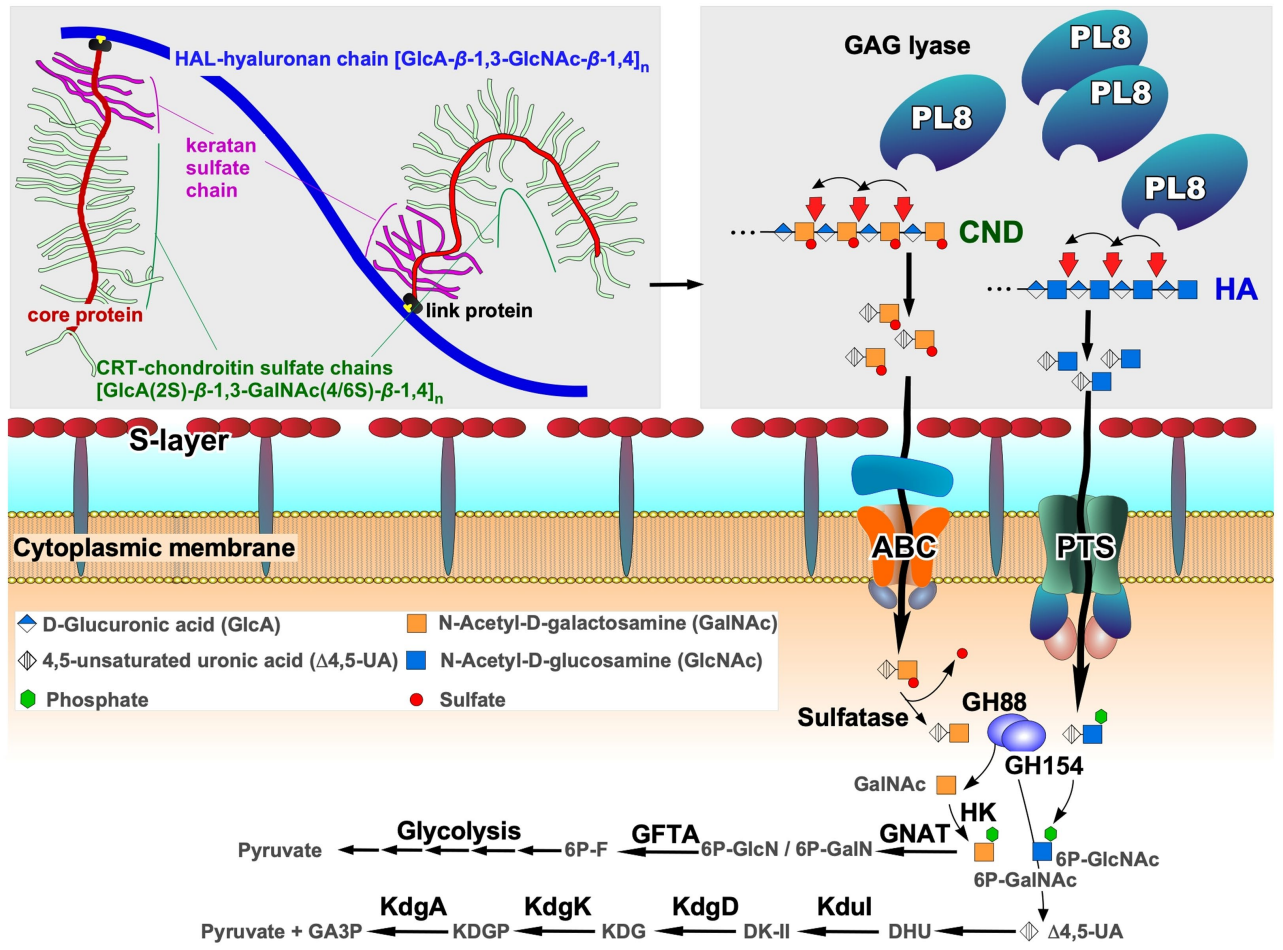
The molecular mechanism of H-hyl isolates’ utilization of hyaluronate and chondroitin, the main components of proteoglycans such as aggrecan (shown as an example), was established based on genome analysis. All H-hyl1, H-hyl2, and H-hyl3 isolates contain these enzymes. Substrate abbreviation used: DHU, 4-deoxy-L-*threo*-5-hexosulose-uronate; DK-II, 3-deoxy-D-*glycero*-2,5-hexodiulosonate; GA-3-P, glyceraldehyde-3-phosphate KDG, 2-keto-3-deoxy-D-gluconate; KDGP, 2-keto-3-deoxy-6-phosphogluconate; 6P-F, fructose-6-phosphate. Enzyme abbreviation used: ABC, disaccharide-specific ABC transporter; HK, hexokinase; KdgA, 6-phospho-2-keto-3-deoxygluconate aldolase; KdgK, 2-keto-3-deoxygluconate kinase; KdgD, 2-keto-3-deoxygalactonate reductase; KduI, 2-keto-3-deoxygalactonate isomerase; GFTA, glutamine:fructose-6-phosphate aminotransferase; GNAT, N-acetylglucosaminyltransferase.

Analysis of the H-hyl genomes revealed the complete genomic repertoire required for conversion and utilization of glycosaminoglycans, such as chondroitin sulfate (CND) and hyaluronic acid (Figure 6). Following the mechanism of action studied with bacterial GAG_lyases (Li and Jedrzejas, 2001; Stern and Jedrzejas, 2006; Jedrzejas, 2007; Sorokin et al., 2025), depolymerization of hyaluronate and chondroitin A by Hhyl isolates likely occurs via an exolytic β-elimination mechanism, resulting in the formation of the corresponding disaccharides consisting of unsaturated glucuronate (Δ4,5GlcUA) and (sulfated) amino sugars. These dimers are subsequently transported into the cytoplasm either by the phosphotransferase system (PTS) or by a specialized ABC disaccharide transporter, encoded in the aforementioned PUL (Figure 5). In the case of PTS, carbohydrate substrates are phosphorylated during passage through the cytoplasmic membrane. The disaccharides are then cleaved into their constituent monosaccharides by unsaturated glucuronidases of the glycosyl hydrolase families GH88 and GH154. Desulfation of CND disaccharides Δ4,5GlcUA*β*(1 → 3)GalNAc-4S by sulfatases likely occurs earlier of this process, as proposed for *Streptobacillus moniliformis* by Oiki et al. (2017). The resulting unsaturated glucuronate is metabolized to pyruvate and glyceraldehyde-3-phosphate through the Entner-Doudoroff pathway by actions of isomerase, reductase, kinase and aldolase, while the phosphorylated amino sugars are metabolized to fructose-6-phosphate, which then enters the glycolytic pathway via a degradation pathway well described for chitin-degrading halo(natronoarchaea) (La Cono et al., 2020; Tulenkov et al., 2025).

### Environmental occurrence

This study provides further evidence that haloarchaea represent a unique group of extreme halophiles with immense metabolic capabilities, representing a massive, largely unknown reservoir of functional diversity and ecological roles. As noted above, the ability of three haloarchaeal isolates to grow and thrive on GAGs, discovered and analyzed in detail, is unparalleled among the so far characterized cultured archaea. This was also demonstrated by a search for similar archaeal lyases deposited in the public database, which revealed nothing but only two similar putative lyases, although belonging to the same family PL8, but most likely active against polysaccharides other than HA and CND. Thus, to determine the ecological distribution of GAG-degrading haloarchaea worldwide, enrichment cultures were additionally established using freshly collected samples available in the laboratory at that time, namely, natural brine and sediments from Samouco solar salterns, Alcochete, Portugal (SSP enrichment) and freshly harvested salt from Cam Ranh field, Vietnam (VSALT enrichment). Both enrichments were scored positive during the growth on HA as the only source of carbon and energy and the metagenomic analysis along with consequent assemblage and annotation revealed the presence of contigs harboring the GAG-degrading PULs, strikingly similar to that of *Natronoarchaeum* sp. H-hyl1 (Figure 1).

Notably, along with the H-hyl1-like PUL SSP1, another, rather unique PUL (SSP2) was found in the Samouco enrichment culture. In addition to a minimal set of GAG-degrading enzymes (GAG_lyase, sulfatase, GH88, and GH154) and an ABC disaccharide transport system, this PUL also contained a glycoside hydrolase of the GH2 family, a large family of hydrolases that include both β-glucuronidase (EC#3.2.1.31) and β-galacturonidase (EC#3.2.1.-). Whereas the SSP2 GAG_lyase was overall very similar to our H-hyl enzymes, it had an additional type I dockerin domain at the C terminus, which is likely involve in substrate binding, as has been proposed for β-1,4-mannan utilizing natronoarchaeon *Natronoglomus mannanivorans* (Sorokin et al., 2024a). Although this is beyond the scope of this study, the isolation of a haloarchaeon with the SSP2 PUL is planned for the future.

## Conclusion

This study presents a detailed characterization of three strains of extremely halophilic archaea and two enrichment cultures obtained from hypersaline lakes and salt production fields across Eurasia, from Portugal to Vietnam. Strains H-hyl1, H-hyl2, and H-hyl3 are aerobic heterotrophs capable of utilizing glycosaminoglycans (GAGs) such as chondroitin sulfate and hyaluronate as their sole carbon (as well as nitrogen?) and energy sources. Phylogenetic analysis based on comparison of 16S rRNA gene sequences and conserved protein sequences placed the new isolates in the genus *Natronoarchaeum* (H-hyl1) and the genus *Haloarcula* (H-hyl2 and H-hyl6) as new-species lineages. This type of metabolism was unknown for haloarchaea (any cultivated members of the Archaeal Kingdom) prior to this study. In addition to cultivation and physiological analysis, further study of the genomes of these archaea allowed us to reconstruct a putative metabolic pathway for GAGs utilization, which potentially allows representatives of these genera to thrive on typical animal acidic polysaccharides, a rather specific polymer uncharacteristic of hypersaline ecosystems. In conclusion, a very important observation should be noted: although GAG-degrading haloarchaea were unknown prior to our study, all five of our attempts to enrich this type of microorganism from natural samples collected across Eurasia, from Portugal to Vietnam, yielded positive results. So, once again, this stresses the fact that a properly chosen method for isolating previously unknown target microorganisms can be quite successful.

## Data Availability

All data used and analyzed in this study have been included in the present article and its Supplementary files. All whole genome and metagenome sequencing information were deposited in NCBI GenBank database and are freely available through the NCBI data under the corresponding BioProject, BioSample, and accession numbers. The assembled genome sequence of Natronoarchaeum rubrum H-hyl1 was submitted to NCBI (BioProject PRJNA1332976; BioSample SAMN51785343). This Whole Genome Shotgun project has been deposited at DDBJ/ENA/GenBank under the accession JBRKBG000000000. The version described in this paperis version JBRKBG010000000. The assembled genome sequence of Haloarcula sp. H-hyl2 was submitted to NCBI (BioProject PRJNA1333890; BioSample SAMN51818556). This Whole Genome Shotgun project has been deposited at DDBJ/ENA/GenBank under the accession JBRIIJ000000000. The version described in this paper is version JBRIIJ010000000. The assembled genome sequence of Haloarcula sp. H-hyl6 was submitted to NCBI (BioProject PRJNA1333891; BioSample SAMN51818726). This Whole Genome Shotgun project has been deposited at DDBJ/ENA/GenBank under the accession JBRIIK000000000. The version described in this paperis version JBRIIK010000000. The metagenome sequencing data of the hyaluronate-degrading enrichment VSALT was submitted to NCBI (BioProject PRJNA1406516; BioSample SAMN54773696). This Whole Genome Shotgun project has been deposited at DDBJ/ENA/GenBank under the accession JBUBAT000000000. The version described in this paper is version JBUBAT010000000. The metagenome sequencing data of the hyaluronate-degrading enrichment SSP was submitted to NCBI (BioProject PRJNA1406516; BioSample SAMN54773624). This Whole Genome Shotgun project has been deposited at DDBJ/ENA/GenBank under the accession JBUBAS000000000. The version described in this paper is version JBUBAS010000000.

## Footnotes

1
https://www.geneious.com

2
https://gtdb.ecogenomic.org/

3
https://enve-omics.ce.gatech.edu/aai/index

4
https://ggdc.dsmz.de/ggdc.php

5
https://services.healthtech.dtu.dk/services/SignalP-6.0/

6
https://bcb.unl.edu/dbCANPUL/
