# Supplementary material for: The degradation of glycosaminoglycans by haloarchaea is apparently a common feature in hypersaline habitats worldwide
Source: Front Microbiol. 2026 May 29;17:1846936. doi: 10.3389/fmicb.2026.1846936 (PMC13260240; doi:10.3389/fmicb.2026.1846936)
Supplement: Supplementary file 1 [file Supplementary_File_1.docx]

**SUPPLEMENTARY INFORMATION**

**Dimitry Y. Sorokin et al.**

**The degradation of glycosaminoglycans by haloarchaea is apparently a common feature in hypersaline habitats worldwide.**


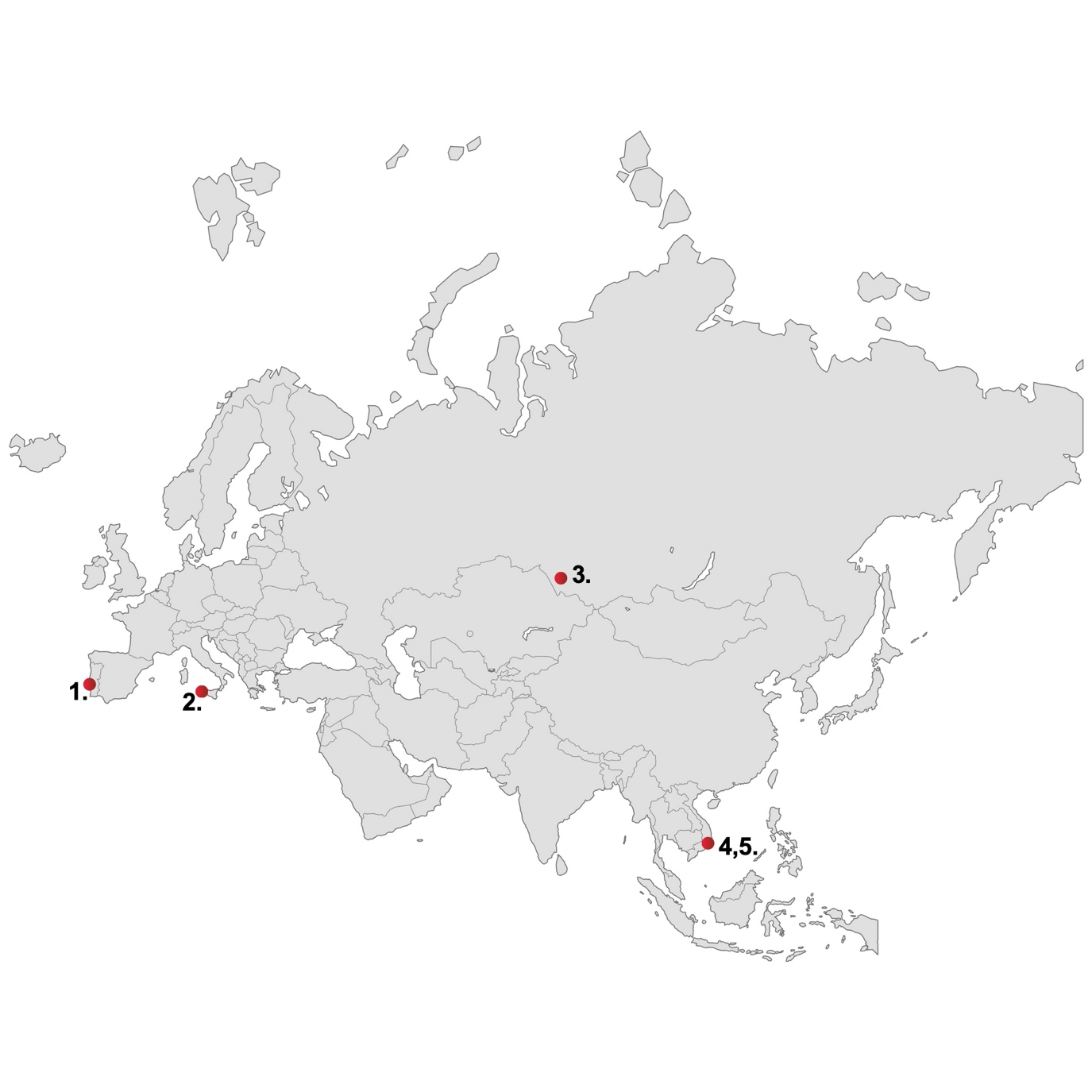


**Figure S1.** World map showing the locations of hypersaline habitats in Eurasia where glycosaminoglycanolytic haloarchaea belonging to the genera *Haloarcula*, *Halorhabdus,* and *Natronoarchaeum* have been isolated in pure cultures or enriched: 1. Samouco solar salterns, Alcochete, Portugal (N 38.7356635 E -8.9976888); 2. Trapani solar salterns, Western Sicily, Italy (N 37.980528 E 12.495000); 3. Kulunda Steppe hypersaline lakes, Altai, Russia (N 51.690000-750000 E 79.720000-88000); 4. Hon Khoi salt fields, Nha Trang, Vietnam (N 12.539860 E 109.208740); 5. Freshly harvested salt, Cam Ranh, Vietnam (N 11.921439 E 109.159131).


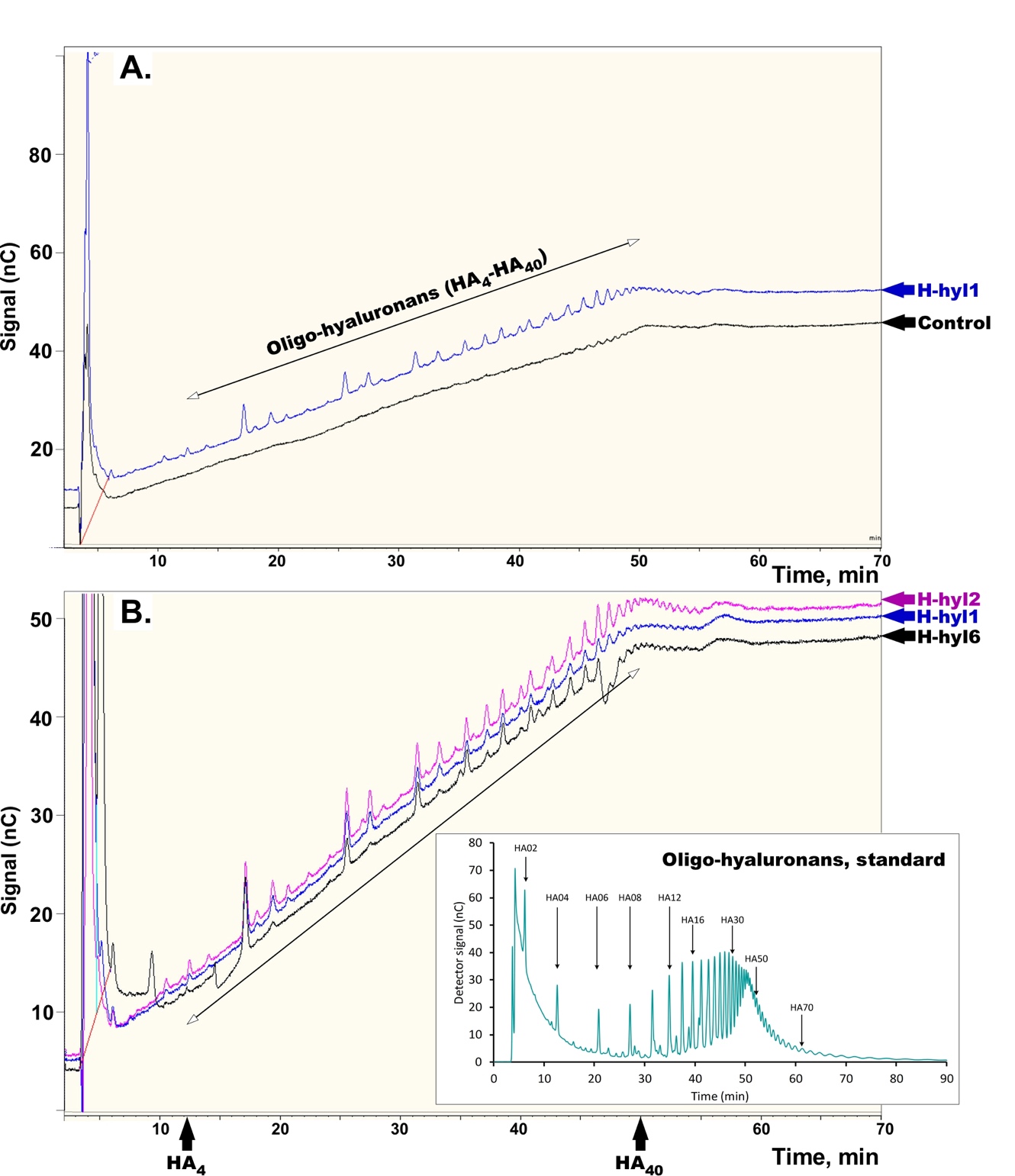


**Figure S2.** Detection of numerous oligo-hyaluronans in the supernatant of a 20-days culture of H-hyl1 by high-performance anion exchange chromatography (HPAEC) compared to an abiotic control of 50 KDa HA exposed for the same period of time (A). HPAEC of the supernatant of the hyaluronate-degrading H-hyl1, H-hyl2 and H-hyl6 cultures (B). Standard oligo-hyaluronans HA_2_-HA_70_ are shown in the inset below on the right.
